# A fresh look at how ocean waves and sea ice interact

**DOI:** 10.1098/rsta.2017.0342

**Published:** 2018-08-20

**Authors:** Vernon A. Squire

**Affiliations:** Department of Mathematics and Statistics, University of Otago, PO Box 56, Dunedin 9016, New Zealand

**Keywords:** ocean waves, sea ice, attenuation, parametrization, power law fluid

## Abstract

Because of their capacity to alter floe size distribution and concentration and consequently to influence atmosphere-ocean fluxes, there is a compelling justification and demand to include waves in ice/ocean models and earth system models. Similarly, global wave forecasting models like WAVEWATCH III^®^ need better parametrizations to capture the effects of a sea ice cover such as the marginal ice zone on incoming wave energy. Most parametrizations of wave propagation in sea ice assume without question that the frequency-dependent attenuation which is observed to occur with distance *x* travelled is exponential, i.e. *A* = *A*_0_ e^−*αx*^. This is the solution of the simple first-order linear ordinary differential equation d*A*/d*x* = − *αA*, which follows from an Airy wave mode ansatz 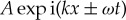. Yet, in point of fact, it now appears that exponential decay may not be observed consistently and a more general equation of the type d*A*/d*x* = − *αA*^*n*^ is proposed to allow for a broader range of attenuation behaviours should this be necessary to fit data.

This article is part of the theme issue ‘Modelling of sea-ice phenomena’.

## 1. Introduction

Ocean wave propagation into and within sea ice fields is a well-established geophysical research topic that is currently attracting renewed attention, prompted by recent adjustments to Arctic sea ice especially, which are occurring as a result of global climate warming. Indeed, reviews [1,2] include exhaustive bibliographies that catalogue early progress in the field, with commentaries from the heroic era of exploration, experimental studies in the 1930s, the introduction of mathematical sophistication in the 1950s and late 1960s, the influential work of Wadhams and others in the 1970s and 1980s [3,4]—the latter prompted by the MIZEX campaign, and more recent further developments using powerful theoretical and numerical solution methods for both shore fast sea ice [5] and the modestly sized ice floes of the marginal ice zone (MIZ) [6].

As well as observations collected *in situ*, remote sensing has also provided valuable datasets using both satellites and aircraft missions. Whether focused upon continuous pack ice, the loosely compacted ice floes of the MIZ, or pancake ice, nilas, frazil and grease ice, the compilation of research papers and reports is impressive and continues to grow as the contemporary importance of the subject is recognized and technological advances make practicable more sophisticated measurements that were not feasible 20 years ago.

Ocean waves propagating in an ice field are observed to decrease in amplitude. The observed reduction is due to a combination of two processes—scattering and dissipation, which both need to be accommodated in any earth system model, ice/ocean model or wave forecasting parametrization. Scattering redistributes energy but does not eliminate it while dissipation, insofar as the waves are concerned, removes energy. Undoubtedly, the latter process actually reassigns the energy to other parts of the atmosphere/ice/water system, e.g. to kinetic energy in the mixed layer, etc., and this will be important in earth system models which are required to conserve energy because they compute results over very long time scales. However, this prerequisite is not important here, as the system under consideration is not closed.

The energy transport equation, or the wave action equation in WAVEWATCH III^®^ (hereinafter WW3) where ocean currents are included [7], is used to embed ocean waves in these large-scale models. We express this equation in its simplest notional form for energy density *E* = *E*(***x***, *ω*, *θ*),
1.1
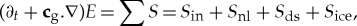
where ***x*** denotes the spatial coordinates, *ω* is the radian frequency (=2*πf* = 2*π*/*T*, where *f* is the frequency and *T* is wave period), *θ* is the direction of travel of the wave, the group velocity *c*_g_ is taken as constant and 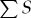 encapsulates a number of source/sink terms, as follows. *S*_in_ represents wind–wave interaction, *S*_nl_ is a nonlinear wave–wave interaction term, *S*_ds_ is a dissipation (whitecapping) term and *S*_ice_ = *S*_ice_(**x**, *ω*, *θ*) is the term of interest in this work as it characterizes how the waves are affected by the ice field. *S*_ice_ can be partitioned into the two processes introduced above, which are, respectively, designated energy attenuation coefficients *α*_scat_ and *α*_dis_,
1.2
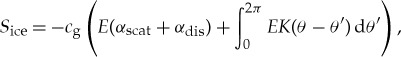
such that 
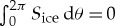
 and 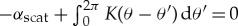 when *α*_dis_ = 0.

The author has specifically referred to *α*_scat_ and *α*_dis_ as *energy* attenuation coefficients, in keeping with [7]. For clarity, I will use the generic unsubscripted symbol *α* to denote the *amplitude* attenuation coefficient later, recognizing that for exponential attenuation *α*_scat_ (and *α*_dis_) ∼2*α*.

Considerable modelling work has been done to understand *α*_scat_ in the MIZ, e.g. the phase-resolving, two-dimensional scattering theory described in [8,9] and tested against field data in [10]. The most appreciable scattering occurs when floe diameters and ocean wavelengths are of similar order, so waves passing through a field of pancake ice will not be scattered to any extent. Although it is recognized that such scattering models are never perfect, the author feels that the redistribution of wave energy that occurs due to scattering which results in attenuation of the wave field expressed through the coefficient *α*_scat_ is well understood and adequately modelled. This is important because contemporary phase-resolving scattering models provide a direct link to ice floe break-up and hence to how the floe size distribution changes, as flexural stresses in the compliant ice floes that make up the MIZ are easily calculated [9]. It only remains to find a numerically efficient way to replicate the properties of *K*(*θ* − *θ*′) in equation (1.2).

Unfortunately, the author cannot say the same in regard to *α*_dis_, for which no satisfactory models exist. Dissipation is due to an abundance of processes that are symptomatic of a MIZ, defined as being the region of the ice cover that is substantially affected by open ocean processes. As such, we expect considerable turbulence; inelastic ice floe collisions that can include reducing the ice to a slurry by pummelling; vortex shedding; wave breaking, overtopping and repetitive overwashing of the floes; spontaneous green water at large wave amplitudes; energy loss associated with ice deformation under extreme conditions; ridging, rafting and ice floe fracture; and no doubt other less obvious mechanisms. Near the ice edge, when the seas are rough, considerable destruction of the ice floes can occur but, because the dissipation eliminates higher frequency waves before lower frequency ones, the zone of intense energy loss is typically limited to 10 or 20 km or so in Arctic waters, see e.g. [11]. In the Southern Ocean, where seas are much fiercer, the zone of destruction will be considerably broader. It is quite likely that the attenuation rates in these outer zones may deviate significantly from those in the interior and may even have a different functional dependence on the distance travelled by the waves. It is also conjectured that large amplitude waves may attenuate differently from those with smaller amplitudes, as the effects of nonlinear dissipation mechanisms such as overwash and wave breaking will be more pronounced.

Creating a viable model that fits the multifarious realizations of the MIZ is unlikely to be achievable, as the contribution from these several dissipative mechanisms will change with both the wave and the ice conditions. Moreover, although the scattered fields are reasonably well understood as noted above, potentially very energetic dissipation will also occur in the waters between floes as a result of the scattering process itself. The convenience of separating *α*_scat_ and *α*_dis_ in equation (1.2) may be problematical in this regard, although we accept its utility. Nonetheless, while its magnitude may change according to ice conditions, in field observations, a simple power law of modest order appears to describe consistently how attenuation varies with wave frequency. To the author's knowledge, no dissipative model has reproduced this proportionality yet unfortunately; indeed most are way off the mark and it is unlikely that a linear model will ever accurately reproduce what is observed.

The above comments notwithstanding, it is conspicuous that the vast bulk of theoretical models constructed to describe how waves are affected by sea ice or vice versa, are configured to fit a linear paradigm, i.e. they employ an Airy wave mode ansatz [12] of the form 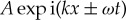. In this expression, *A* is the initial wave amplitude, *k* denotes a generic complex wavenumber that here defines either propagation in the water or beneath the ice cover, *x* is the direction of propagation and *t* is time. In the usual way, *k* = *κ* + i*α* encapsulates dispersion (via the real quantity *κ*) and attenuation (via imaginary i*α*) into one consolidated complex wavenumber. Typically, a boundary value problem is then solved, e.g. for open water surface gravity waves travelling into an homogeneous plate or layer which can be semi-infinite or finite in horizonal extent and has prescribed physical properties that define its behaviour in flexure. As a rule, the ice would be designated as elastic, viscous or viscoelastic; each being reasonable when the wave amplitudes are modest for the strain rates induced by typical surface waves in the sea ice under various circumstances. The material properties chosen for the ice provide the dispersion relation that regulates how the waves propagate under the ice cover, i.e. how they disperse and reduce in amplitude, and, because the wavenumbers in open water and ice-covered sea are different, the impedance change at the ice edge which causes some of the wave energy to be reflected. Weakly nonlinear formulations exist but they are relatively rare, e.g. [13].

The author is not dismissing linear models that describe wave propagation into and within ice fields. With graduate students and colleagues, I have constructed no end of linear, physically based models, e.g. [5,6,8,9], over many years. Rather, I am cautioning that the *a priori* adherence to the linear *A* = *A*_0_ e^−*αx*^ prototype can be problematical when (i) parametrizing in operational forecasting systems and earth system models, as it predisposes the attenuation to be exponential; (ii) attempting to fit any such model to data, because the exponential function *A* = *A*_0_ e^−*αx*^ may have too few degrees-of-freedom to fit the data at all the spectral wave frequencies present. Moreover, (ii) may contribute to enigmatic intermittent features that have been seen in some field datasets, e.g. [4], acknowledging that specific phenomena such as ‘rollover’, whereby attenuation peaks at some wave frequency but decreases again at higher frequencies, has recently been explained by wind input and nonlinear energy transfer between frequencies [14].

## 2. Appraisal

### (a) Two paradigms

A crucial distinction needs to be made between two classes of theoretical model, designated paradigms I and II herein. Papers [5,6,8,9] are paradigm I examples of models where the physics of wave–ice interaction is replicated theoretically as credibly as possible. In [5], the reflection and transmission coefficients are found at the interface between an open water half-space and a half-space covered by a uniform ice sheet that represents so-called shore fast sea ice. In concert with many models describing how ice flexes in response to waves, the ice is assumed to respond as a thin elastic plate. Paper [6] also computes reflection and transmission coefficients, but this time for a finite elastic ice floe. In the third example [8], a MIZ bathed by a prescribed directional wave spectrum is modelled by means of large numbers of floating compliant plates which scatter the penetrant wave energy in all directions. The fourth example [9], leads on from [8], using its scattering theory to evolve the MIZ floe size distribution, by breaking up those ice floes that are too large to exist in the wave field using a Mohr–Coulomb fracture criterion. In all cases, the physical properties of the ice, namely observable state variables such as thickness, ice density and the elastic moduli, etc., can be mapped straightforwardly onto the coefficients that appear in the model and *in situ* experiments can be done to ascertain whether the model is a good fit to data. It is these attributes that are symptomatic of a paradigm I model.

On the other hand, some theoretical work is more accurately labelled as a parametrization and fits paradigm II, including models that are constructed for one purpose being used for another. I am not dismissing the value of paradigm II, as it is simply not practicable to incorporate fully phase-resolving wave–ice interaction theory into either an earth system model or WW3; a pragmatical solution is, therefore, necessary that parametrizes the physics in the most accurate way. Potential examples in current use are the viscoelastic layer in [15], which is well suited to modelling waves travelling in homogeneous continuous ice, or the modified fast ice model [5]^*μ*^ altered to have a complex flexural rigidity so as to produce damping, being used to model an entire, potentially open, i.e. of low-concentration, heterogeneous ice field. (The superscript *μ* denotes viscosity, added to acknowledge that I am referring to a viscoelastic version of the original elastic paper [5].) Parametrization aspires to represent a substantial region of ice cover composed of many ice floes and ice cakes present at spatially variable concentrations and thicknesses as an *effective* medium with a single dispersion relation that describes how the waves disperse and attenuate via their wavenumber as they propagate. Zones with different physical properties can be introduced, recalling that the impedance alters where properties change so that a boundary value problem exists at each interface which strictly requires reflection and transmission coefficients to be found. However, the real challenge is mapping a loosely configured, heterogeneous array of independent ice floes, ice cakes and frazil onto the physical material coefficients that appear in the ‘holistic’ dispersion relation that describes the effective medium. Unlike when the examples [5,6,8,9,15] given above are used as originally intended, there is no way that this can be done by independently measuring each physical property of the sea ice and the only recourse is to measure how the waves change as they pass through the ice medium and then to tune the model parameters to ‘best fit’ the observed data. Accordingly, the model is being calibrated with the very data it is predicting.

The computed model parameters also strictly have no material physical interpretation as they are only associated with the experimental data being analysed, so generalization to other ice fields or ocean wave states is challenging or impossible. Moreover, it must be asked how faithful the model is to the physics of the process being observed and whether it is actually capable of replicating observations. The author contends that this has not been convincingly demonstrated to date. Indeed, there are incontestable analyses that suggest that [5]^*μ*^ and [15] can never replicate observations well as a paradigm II stratagem because their asymptotic behaviour at mid- to high-frequencies is usually wrong and the same is true of viscous layer models such as that in [16].

Without knowing that they are a universally valid parametrization, some prototype models are already being embedded within operational wave forecasting systems such as WW3 and earth system models that operate under a very diverse range of environmental circumstances, e.g. for large wave steepnesses where the differential equation predicting attenuation, namely d_*x*_*A* = − *αA* with solution *A* = *A*_0_ e^−*αx*^, is unlikely to be a reliable approximation to Nature in all circumstances and the issue of poor adherence to observed *α*(*ω*) behaviour at high frequencies is becoming clear. (Here and subsequently the abbreviation d_*x*_≡d/d*x* is used.)

Although the purpose of this paper is not to review the field of wave–ice interactions, it would be remiss of me not to corroborate the assertions I have made as best I can. This will be done in later sections. Subsequently, using data from a recent field experiment, I will focus on the constraints implicit in linear models that conflate dispersion and attenuation in ice fields using the single complex wavenumber *k* = *κ* + i*α*. An alternative differential equation, namely d_*x*_*A* = − *αA*^*n*^, derived using physical arguments for pancake ice in [17] and potentially having an additional degree-of-freedom *n*, will also be presented as a generalization of the archtypal exponential attenuation law *A* = *A*_0_ e^−*αx*^. Remarkably, the model in [17] affords the same equation for attenuation built from different physics in [18]. While the physical basis of the latter publication can be challenged, the differential equation that parametrizes how wave amplitude *A* changes from its value *A*_0_ at the ice edge as the wave advances through the ice medium, namely
2.1
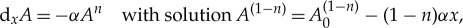
where *n* and *α* are to be found, is a generalized decay law that reverts to one with a constant rate of decay when *n* = 0 and exponential decay when *n* = 1 [17,18]. The equation is a consequence of allowing viscosity to depend on strain rate and, because of this, frequency *ω*, in a particular way and derives from a power law fluid constitutive relation. Rather than just ‘best fit’ observations, I will also describe how the value of *n* can be predetermined to some extent by aggregating intelligence about the nature of wave–ice interaction in MIZs.

Irrespective of the simplicity of 2.1 and the cognate linear equation when *n* = 1, I reiterate that ice fields are never homogeneous. Accordingly, it is highly unlikely that *any* parsimonious decay equation will replicate the behaviour of ocean waves travelling in sea ice perfectly, because the attenuation experienced depends on the local oceanographic and ice properties as reported in [11] for the Bering Sea. In principle, zones with different values for *n* may help but, recognizing the immense challenge of conducting wave experiments in sea ice, the importance of comprehensive sea ice morphology and oceanographic observations collected simultaneously to complement the wave data cannot be overstated if we are serious about calibrating an aspiring parametrization.

### (b) Contemporary parametrization

I briefly discuss the three most common material descriptions used to categorize sea ice when it is subjected to ocean wave forcing; elastic, viscous and viscoelastic. The common theme is (i) linearization about the basic state of rest, (ii) assuming the motion is proportional to 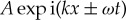 and (iii) derivation of a dispersion equation that connects *ω* with *k*. The dispersion relations, *ω* = *ω*(*k*), are distinct in each case and for different models but the dependency of the amplitude *A* on *x* that follows is always exponential.

The generic ice wavenumber *k* is real when the ice is uniform and purely elastic if no further dissipation, arising from a combination of ice flexing, dissipation in the water and mechanical energy loss such as collisions between ice floes or cakes, is parametrized. Waves disperse differently under solid ice compared with open sea and also attenuate due to scattering. This is because the ice-coupled wavenumber depends on the physical properties of the sea ice so transitions of ice morphology, e.g. thickness, or the edges of ice floes cause reflections to occur. However, this process represents a redistribution of energy as opposed to dissipation. In the context of this paper, this means that a perfectly elastic sea ice cover can only reduce wave amplitude if the ice is not spatially uniform.

A number of papers represent the entire sea ice cover as a viscous layer at the ocean surface, e.g. [16] models wave propagation in a Newtonian viscous layer floating on an inviscid ocean, while [19] add mathematical complexity of indeterminate oceanographical usefulness by making the underlying ocean viscous. The primary focus of the model in [16] was to explain some laboratory observations on wave propagation in grease ice written up subsequently in [20], whereas a broader range of ice types including the MIZ are included in [19]. Using a Lagrangian formulation, Weber [21] employs a viscous model also to represent a MIZ—in this case for an unlayered, rotating ocean. A viscous boundary layer model based upon the eddy viscosity in the turbulent boundary layer beneath the ice cover has also been suggested [22–24], recognizing that eddy viscosity is a phenomenological parameter to be determined as a function of flow conditions rather than a physical measurable viscosity. These few papers collectively illustrate a reasonably common way of parametrizing the aggregated effect of sea ice on waves.

In fact, sea ice itself is viscoelastic, with different degrees of nonlinearity dependent on its physical properties and environmental circumstances. At ocean wave forcing frequencies, first-year sea ice itself is approximately anelastic [25], i.e. any viscous deformation is recoverable, which is a specific form of viscoelasticity where the hysteresis loop is closed. However, current viscoelastic plate [26] and viscoelastic layer [15,27–29] models—the latter originally built to synthesize the elastic plate [5] and viscous layer model [16], ignore this subtlety. Instead, they accommodate other kinds of sea ice deformation and/or alternative dissipative mechanisms that cause energy loss as the waves propagate into and through the material. In doing this, they are including both the modest dissipation due to sea ice flexure plus the typically substantial energy loss arising from the several known but neglected pervasive mechanisms discussed in §1—many of which are nonlinear but are being rendered in a linear way in the model. In all cases, a dispersion relation *ω* = *ω*(*k*) results that, as usual, is constructed by first assuming the Airy wave mode ansatz 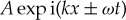 with *κ* = Re *k* expressing dispersion and *α* = Im *k* expressing attenuation via *A* = *A*_0_ e^−*αx*^ so any nonlinearity is neglected *a priori*.

It is also noteworthy that variations in concentration *c* are treated by reducing the effective medium's response linearly. At first sight this seems reasonable but, when it is recognized that most of the dissipation arises because of turbulence in the water, it becomes counterintuitive. Surely the level of dissipation would increase initially with a reduction in *c*, as ice floes become more mobile and ‘belligerent’, and then start to decrease as *c* → 0 as the effects of floe collisions, waves breaking over floes and overwashing subside.

Observations suggest that *α*(*ω*)∝*ω*^2^–*ω*^3^ in many circumstances, yet Meylan *et al.* [30] show, after switching off any elasticity, that [5]^*μ*^ and [16] have power 11 and power 7 asymptotic proportionality, respectively. The dispersion relation in [15] is so complicated that it is hard to be absolutely sure about its asymptotic behaviour but with zero shear modulus it is expected to behave like the Keller model [16], so is also likely to be power 7. Enhanced viscosity ocean models [21–24] go as *ω*^7/2^ when dispersion is assumed to be the same as in open water. The closest to *ω*^2^–*ω*^3^ to date is the model in [31], for which *α*(*ω*)∝*ω*^3^. It has simple velocity-dependent damping incorporated in the elastic plate model [5] as an alternative to the flexural rigidity being made complex in [5]^*μ*^.

**Figure 1.**
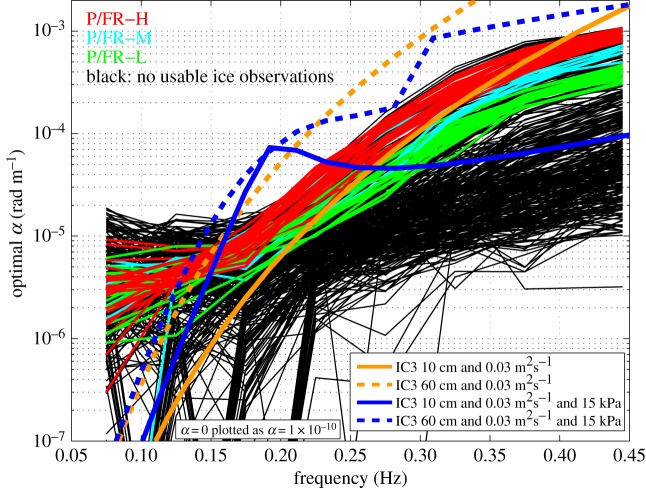
Dissipation profiles from a simple application of the Wang and Shen model [15] are shown in orange (without elasticity) and blue (with elasticity) for two ice thicknesses (solid: 10 cm; dashed: 60 cm), alongside field data from the 2015 R/V *Sikuliaq* Beaufort Sea field experiment. After [32], with thanks to Dr Erick Rogers.

This problem is well illustrated in figure 1, which shows attenuation coefficient *α* plotted as a function of wave frequency *f* from 403 wave buoy spectra collected during a 2015 field experiment in the Beaufort Sea carried out from the ship R/V *Sikuliaq* in a mixture of pancake ice and frazil. Results are clustered into three coloured sub-groups representing experiments where dissipation was high (red), medium (cyan) and low (green). When no usable ice observations are available the experiment's *α* are shown in black. Using an inversion process to provide physical moduli, these data are compared with the viscoelastic layer model [15] with the shear modulus set to zero (orange)—making it equivalent to the viscous layer model in [16], and with non-zero shear modulus (blue); each for two ice thickness values. Figure 1 indicates that the steepness of the curves is notably greater than that implied by the inversion process but that the introduction of elasticity reduces the steepness. Unfortunately, including elasticity also causes root finding difficulties [26]. Presumably, *α*(*ω*)∝*ω*^7^ behaviour contributes to the observed steepness. A more general equation describing attenuation such as d_*x*_*A* = − *αA*^*n*^ could possibly help, as we shall find that it allows viscosity to be functionally dependent on frequency as opposed to being constant (see §3).

## 3. The power law model

### (a) A granular floe jostling model

To the author's knowledge, the differential equation d_*x*_*A* = − *αA*^*n*^ was first applied to sea ice back in 1973 [18], specifically invoking a model of dissipation based on the Glen–Nye flow law for glacial steady-state flow, where *n* = 3. In its general form the equation requires that the viscosity of the deforming material *μ* is not constant, as it would be for Newtonian flow, but that it depends on strain rate. When *n* = 3, *μ*( · ) is inversely proportional to the square of 
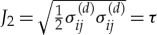
, which is defined as the second invariant of the deviatoric stress tensor *σ*^(*d*)^_*ij*_, but when *n* is unspecified *μ*( · )∝*τ*^(1−*n*)^. The quantity *τ* is called the effective shear stress or octahedral stress. Physically, a choice of *n* = 3 is hard to justify as anything other than a parametrization characterizing the synthesis of all of the dissipative processes effected on ocean wave trains as they propagate within a MIZ, because there is no physical argument that can explain why waves should lose energy in the same manner as a creeping glacier. The strain rates associated with the two natural phenomena are different by several orders of magnitude. Yet, despite the disputable time scales, the model did surprisingly well in replicating observations in both the Arctic and Antarctic MIZs for the particular wave fields and ice covers that prevailed at the time [18].

Nonetheless, although serendipitous, back-of-the-envelope calculations of pancake ice subjected to waves invoking granular flow theory [17], produce the same differential equation, i.e. d_*x*_*A* = − *αA*^*n*^. The equation actually arises from the so-called power-law fluid, defined such that
3.1
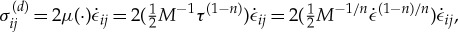
where *M* is constant, *n* > 0 with *n* = 0 treated as a special case, 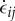 is the strain rate tensor and 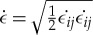 is effective strain rate.

Power-law fluids have a strain-rate-dependent apparent viscosity 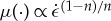. The value that the index *n* takes on determines the way the material deforms; for example, when 0 < *n* < 1, *μ*( · ) increases with increasing strain rate and it is said to be dilatant or shear thickening; when *n* = 1,  *μ* = const.; and when 1 < *n* < ∞, *μ*( · ) decreases as strain rate increases and the material is described as pseudo-plastic or shear thinning. Because shorter period waves reduce in amplitude more rapidly than longer ones in sea ice, i.e. attenuation increases as frequency and strain rate increase, the phenomenon of wave–ice interaction is dilatant. This does not, however, necessarily mean that sea ice is a dilatant material; indeed at very long time scales it would be expected to behave similarly to fresh water glacial ice and to be pseudo-plastic. To the author's knowledge, the power law fluid has not been considered as a viable constitutive relation for sea ice itself.

Interestingly, dilatancy has the special cases (i) *n* = 0, where viscosity *μ* increases at a uniform rate with *τ*, which in the present context leads to the imperative of wave trains reducing in amplitude in direct proportion to the distance they travel and, axiomatically, the complete elimination of wave energy over a finite distance; (ii) 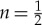, where *μ* increases uniformly with 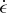 and (iii) *n* = 1, where *μ* = const. and an exponential decay law holds. Of course, *n* can take on other values that determine whether the attenuation is less or more rapid than exponential over a finite domain. Recognize that in a power law fluid *n*≥0, as stated. This is not necessary for equation (2.1), where *n* < 0 can lead to very rapid attenuation of the incoming sea, which increases with distance travelled unphysically for a uniform ice cover.

### (b) Choosing *n*

In §2b, I observed that several models describe wave propagation in the MIZ by making the ocean more viscous there, typically using an eddy viscosity that captures the enhanced internal fluid friction arising from the turbulent transfer of momentum by eddies analogous to the action of molecular viscosity in laminar flow but on a much larger scale. These models have a dispersion relation of the form *μg*^2^*k*^4^ = 2*ω*(*ω*^2^ − *gk*)^2^ and furnish an *α*(*ω*)∝*ω*^7/2^ for a very thin viscous layer overlying an ocean of much lower viscosity (see equations (4.14) and (4.15) of [21]). While this result is only asymptotic, by way of demonstration I ask the question ‘can *μ*( · ) be adjusted such that the order of these viscous models is reduced from *ω*^7/2^ to *ω*^2^ by choosing *n* such that the excessive dilatancy is eliminated to give a beneficial *μ*(*ω*)?’. It is conjectured that *n* = − 2 achieves this goal, ultimately producing *α*(*ω*)∝*ω*^2^ from that predicted by the dispersion relation.

**Figure 2.**
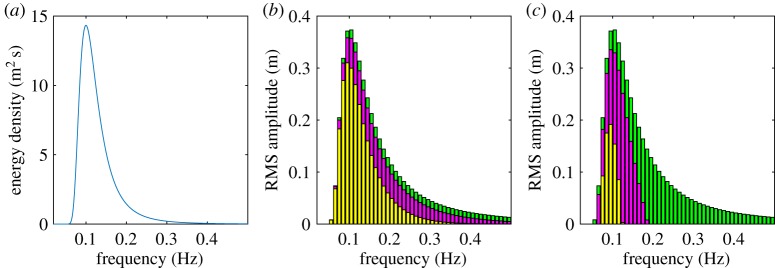
(*a*) The Pierson–Moskowitz spectrum *E*_0_ defined by equation (3.2). (*b*) The partially obscured green bar graph is an RMS amplitude spectrum *A*_0_(*f*) created from *E*_0_(*f*) by integrating across frequency bands of width 0.01 Hz with the central frequency at the mid-point. *A*_0_(*f*) is plotted as a bar graph to emphasize that each amplitude is valid over a frequency band, e.g. from 0.2 to 0.21 Hz with a central frequency 0.205 Hz, rather than at a single frequency. The other amplitude spectra, coloured magenta and yellow, respectively, show how *A*_0_ evolves exponentially, i.e. when *n* = 1, as *x* increases. (*c*) Amplitude spectra constructed in the same manner as for (*b*), but for *n* = 0. Identical values for the constant of proportionality in *α*∝*f*^2^ and the distances from the ice edge are used for (*b*) and (*c*), chosen to emphasize the disparity between the two types of attenuation. It is the relativity between the same colours in plots (*b*) and (*c*) that is important, rather than the absolute values.

Also, by way of illustration, we can investigate how a plausible RMS amplitude spectrum *A*_0_(*f*) evolves with distance *x*, again assuming that *α*(*f*)∝*f*^2^ and recalling that *ω* = 2*πf*. *A*_0_(*f*) is derived from the quintessential Pierson–Moskowitz energy density spectrum *E*_0_ = *E*_0_(*f*) at the ice edge, by integrating across a comb of frequency bands and then taking the square root. What we are calling an RMS amplitude spectrum is, therefore, the square root of a spectrum composed of a set of contiguous bandlimited energy density integrations. Although *A*_0_(*f*) depends on bandwidth, this is unimportant here as all calculations are relative rather than absolute. The Pierson–Moskowitz spectrum *E*_0_, see figure 2*a*, has the form
3.2
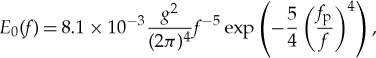
where *f*_p_ is the peak frequency which is set to 0.1 Hz to be consistent with [32], *g* is the acceleration due to gravity and the numerical constant 8.1 × 10^−3^ is known as the Phillips constant [33]. Equation (3.2) can be integrated to give 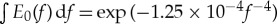, so it is an easy matter to create *A*_0_ directly or by numerical integration. The two sets of amplitude spectra, coloured green, magenta and yellow in figure 2*b*,*c*, shows the frequency-dependent attenuation experienced by the *A*_0_(*f*) spectrum as it advances into the notional ice cover, which either (figure 2*b*) attenuates exponentially with penetration *x*, or (figure 2*c*) in direct proportion to *x*. It is evident that the latter decay (figure 2*c*) is much greater for the same constant of proportionality in *α*∝*f*^2^. Although artificial, it does demonstrate the potential of the more general behaviour furnished by d_*x*_*A* = − *αA*^*n*^, which may be especially useful for parametrizing dissipation caused by the aggregation of nonlinear processes that expunge energy from incoming wave trains for the first 10 or so kilometres from the ice edge or, in all likelihood, farther when wave amplitudes are large (see §1).

Finally, let us consider some real data and arbitrarily adopt the solid red curve in fig. 6 of [32], which shows that the outer energy density *E*_0_(*f*) has an *f*^−4^ tail above *f* = 0.1 Hz, i.e. *E*_0_(*f*)∝*f*^−4^⇒*A*_0_(*f*)∝*f*^−2^. (The Pierson–Moskowitz spectrum has an *f*^−5^ tail and we have confirmed that the *A*_0_(*f*) spectrum in figure 2*b*,*c* has an *f*^−5/2^ tail, as required.) Seek a model that has an *α*(*f*)∝*f*^2^ form irrespective of the value of *n*, interpreting *α* throughout equivalently. Three examples are considered, the first of which is somewhat artificial, as follows.

(i) Suppose that the *f*^−4^ tail in the spectrum doesn't change as the wave field from which it is constituted propagates farther into the sea ice cover. Using equation (2.1), it is straightforward to argue that *n* = 2 is the only value of *n* that can produce the desired behaviour for *α* at frequencies above about 0.1 Hz. For *n* = 2, *α* = (*A*^−1^ − *A*^−1^_0_)/*x*.
(ii) Exponential attenuation is recovered when *n* = 1. In this case, amplitude 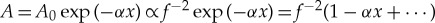, so that 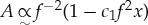 to first order, i.e. the initial decay is proportional to distance covered.
(iii) For *n* = 0, *A* = *A*_0_ − *αx*∝*f*^−2^(1 − *c*_2_*f*^4^*x*).

In the above *c*_1_ and *c*_2_ are constants that arise from the proportionalities assumed, i.e. in the tail of the incident energy density spectrum, where *E*_0_(*f*)∝*f*^−4^ and *α*(*f*)∝*f*^2^. Although *A*_0_ initially reduces in direct proportion to distance *x* when *n* = 1, the decrease with *x* is greater for the *n* = 0 example if the magnitudes of *α* are numerically similar (recalling that the interpretation of *α* and its units differ in each case).

### (c) Limiting cases

Based upon observations transmitted to satellite by some floating wave buoys, it is reported that the significant wave height of modest incident seas diminishes exponentially in the Southern Ocean MIZ [34], but that attenuation proportional to distance travelled is observed when seas are higher. The properties of the ice field through which the waves propagate undoubtedly affect the amplitude at which the transition occurs. A similar analysis of field data from an experiment collected during the 2015 R/V *Sikuliaq* field programme produces an analogous conclusion for pancake ice [35], specifically that significant wave heights less than 3 m seem to reduce exponentially with distance *x* but in direct proportion to *x* when they exceed 3 m. However, respecting the comments I made earlier about ice field heterogeneity, it is acknowledged that it is also possible for these data that this effect is due to the sea ice being more broken up when the waves are higher.

**Figure 3.**
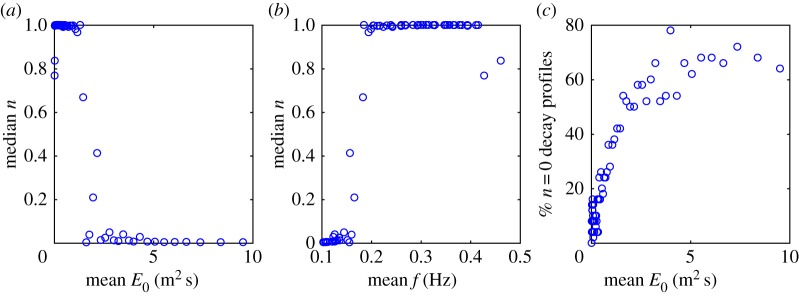
Panel (*a*) shows *n* versus *E*_0_, created from clusters of *E*_0_ that each contain about 100 estimated values. Each point on the plot gives the mean of this sample of *E*_0_ values versus the median of the corresponding sample of *n* values. The transition between *n* = 1 and *n* = 0 occurs at *E*_0_∼1.5 m^2^s. The median of *n* versus the mean of the frequency calculated as the mean of all the frequency samples corresponding to the *E*_0_ samples in a bin is shown in (*b*). There is a dependence on frequency which follows closely the dependence on energy because the two are strongly correlated for *f* > 0.1 Hz in the spectral tail where *E*_0_∝*f*^−4^. (*c*) The percentage of profiles in each *E*_0_ bin where *n* = 0, i.e. the waves decay in direct proportion to the distance traversed.

Reanalysis of the dataset in [34] over two distinct frequency bands without integrating across all frequencies to get significant wave height [36] reaches the alternative conclusion that, although long period swells are insensitive to wave amplitude and consequently behave linearly, the attenuation of short period waves increases with wave amplitude. This outcome is based upon an *a priori* assumption of exponential decay across pairs of stations using the Airy wave mode ansatz introduced earlier. As a consequence, it is of interest to establish whether a similar kind of attenuation occurred during the 2015 R/V *Sikuliaq* field programme, where wave amplitudes were often quite large during one of the experiments. This accords with our original conjecture in §1. We consider the single wave attenuation experiment that took place from 11 to 13 October 2015 in figure 3, where the median 0≤*n*≤1 value extracted from a direct best fit of the SWIFT buoy data [32,35] is plotted against energy density in figure 3*a*, median *n* is plotted against the mean value of frequency in figure 3*b* and the percentage of *n* = 0 attenuation profiles as a function of energy density is plotted in figure 3*c*. (This notwithstanding, note that the more thorough analysis reported in [35] contrasts results from the two types of wave buoy deployed in the experiment.) It is evident that the likelihood of a profile being of type *n* = 0, increases with the energy density and hence wave amplitude from figure 3*a*. From figure 3*b*, *n* = 0 is also associated with low frequencies but this is because the shape of the wave spectrum entering the ice field goes as *f*^−4^ in the part of the spectrum where significant energy exists. The percentage of *n* = 0 decay profiles increases monotonically with energy density, flattening out at about 60% or so beyond an *E*_0_ of about 5 m^2^s as shown in figure 3*c*. While it is acknowledged that these results may be due to variations of ice type as opposed to larger amplitude waves, or indeed to other geophysical variations, the resilient dependency on the magnitude of *E*_0_, the abruptness of the switch from *n* = 1 to *n* = 0, and figure 3*c* add weight to our conjecture. Observe, however, that figure 3 relates to energy density and not to amplitude as expressed in equation (2.1) and that while *n* = 1 implies exponential decay for both *A* and *E*_0_, a decay in *E*_0_ that is directly proportional to *x* does not mean the same for *A*. While equation (2.1) may hold for both amplitude and energy density, the value of *n* can be different.

## 4. Conclusion

The most significant opinions expressed in this paper are drawn together as a useful resource.

(i) It is unfeasible to use a paradigm I model in a global scale earth system model or WW3.
(ii) Ocean waves travelling in sea ice reduce in amplitude due to two phenomena: conservative scattering by ice floes, which relocates energy; and true dissipation, arising from several poorly quantified nonlinear phenomena that remove energy.
(iii) Dissipation is most extreme near the ice edge and/or when the waves are most fierce but the width of the zone over which it dominates other sources of attenuation will vary with the wave and ice conditions.
(iv) Scattering is most significant when floe diameters and wavelengths are of the same order, while dissipation is always present to a greater or lesser degree depending on the properties of the sea ice and the waves.
(v) Although the energy transport equation's source/sink term *S*_ice_, defined in equation (1.2), separates *α*_scat_ and *α*_dis_, the scattered wave field will also be subjected to considerable dissipation that will reduce its impact on surrounding floes.
(vi) Ocean waves break up ice floes to create the floe size distribution, an important process that is modelled by scattering theory, taking into account the flexural stresses in the floes provoked by wave-induced bending [9].
(vii) Nearly, all contemporary models constructed to characterize wave propagation into and beneath sea ice in its several forms are linear including those associated with paradigm II, with some having been reapplied to represent ‘effective media’ in circumstances that they were not originally intended to model.
(viii) The fidelity of current effective media models in the MIZ has not yet been demonstrated.
(ix) Material constants in effective media have no physical meaning, as to make its predictions the medium aggregates a smorgasbord of disparate mechanisms and calibration is unattainable because of the unique set of conditions that define each experiment.
(x) Conceding the scattered nature of the field data, it appears that the attenuation coefficient *α*(*ω*)∝*ω*^*p*^, 2≤*p*≤3, with some experiments suggesting *p* = 2, yet contemporary paradigm II parametrizations do not easily reproduce this asymptotic behaviour.
(xi) Primarily because of dissipation, there is reasonable observational evidence to conclude that attenuation is not always exponential and a natural consequence of this is that waves may experience different ‘attenuation laws’, during their passage through ice fields.
(xii) Because of nonlinearity, exponential attenuation appears to be associated with waves of low amplitude while more substantial waves may experience other kinds of damping, e.g. proportionate to the distance they travel.
(xiii) While at first sight scaling material constants in parametrizations in proportion to concentration is physically plausible, it may actually not be an effective universal stratagem because of the substantial damping that can occur in the water between ice floes.
(xiv) Acknowledging that, as always, calibration will be challenging, the equation d_*x*_*A* =  − *αA*^*n*^ with solution *A*^(1−*n*)^ = *A*^(1−*n*)^_0_ − (1 − *n*)*αx*, or its equivalent energy density form with a different value for *n*≠1, may be helpful as a simple yet more general parametrization of wave attenuation in pancake ice that includes both scattering and dissipation.

## Data accessibility

Data used to generate figure 3 were accessed from http://www.apl.washington.edu/arcticseastate. Nontrivial Matlab codes and data files created for analyses can be accessed through https://www.maths.otago.ac.nz/~fmontiel/Data.
